# IMPROVING HPV VACCINE RATES FOR ABORIGINAL YOUNG PEOPLE THROUGH A SOCIAL MEDIA CAMPAIGN: AN INTERRUPTED TIME SERIES

**DOI:** 10.12688/f1000research.168930.1

**Published:** 2025-10-24

**Authors:** Natalie A. Strobel, Jocelyn Jones, Kim Gates, Simon L. Turner, Joanne E. McKenzie, Daniel R. McAullay

**Affiliations:** 1 Kurongkurl Katitjin, Edith Cowan University, Mount Lawley, Western Australia, Australia; 2 Aboriginal Health Council of Western Australia, Highgate, Western Australia, Australia; 3 Methods in Evidence Synthesis, Monash University School of Public Health and Preventive Medicine, Melbourne, Victoria, Australia

**Keywords:** HPV, Aboriginal and/or Torres Strait Islander Peoples, vaccination, social media

## Abstract

Human papillomavirus (HPV) is a common sexually transmitted infection that can lead to HPV-related cancers such as cervical cancer and other cancers such as anal, vaginal, and penile cancer. HPV rarely produces symptoms and cannot be cured or treated; therefore, vaccination is essential to protect against HPV and HPV-related diseases. However, young Aboriginal and Torres Strait Islander (hereafter, respectfully, ‘Aboriginal’) people are not receiving their HPV vaccine dose, resulting in missed opportunities to be protected from HPV-related cancers.

Health promotion is a critical way to empower people to take ownership over and control their health and is a core function of public health. One way to deliver health promotion is through social media platforms. In recent COVID-19 times, we have seen the effect of social media on public health messaging, both positive and harmful. In particular, Instagram influencers have had a profound impact on pro- and anti-vaccination messages. Aboriginal adolescents aged 13-15 years old have strong engagement with social media platforms. Combining co-created health promotion messages with local ‘micro-Influencers’ is a novel way to improve HPV vaccination rates.

The overall aim of this project is to improve the rates of HPV vaccination amongst young Aboriginal people. We will achieve this through: i) co-creation of health promotion messages, ii) developing and delivering a social media campaign and iii) evaluating the effectiveness of the campaign through an interrupted time-series design. We hypothesise that the co-creation of health promotion messages with young Aboriginal people and their families and utilising the skills of micro-Influencers to engage and influence their followers will result in improvements HPV vaccination rates amongst this population.

## Introduction

Human papillomavirus (HPV) is a common sexually transmitted infection that can lead to HPV-related cancers such as cervical cancer and other cancers such as anal, vaginal, and penile cancer. ^1, 2^ HPV rarely produces symptoms and cannot be cured or treated; therefore, vaccination is essential to protect against HPV and HPV-related diseases.

In 2007, Australia initiated the HPV vaccine through the National Immunisation Program (NIP) to girls aged 12 to 13 years in 2007 and to boys in 2013; catch up programs were implemented for older age groups. Up until 2023, the HPV vaccine provided as a free two-dose program for both males and females aged 12-13 years, with doses given between 5-6 months apart through school-based programs. ^3^ Responding to the global decline in HPV vaccination, lack of access to the vaccine and cost and burden required for two doses, WHO released updated recommendations in 2022 that a single dose was effective in reducing HPV. ^4^ As of 2023, Australia has transitioned to the single dose for 12-13 year olds through the NIP delivered at school with young people up to 26 years old also eligible for the free HPV vaccine. If a vaccine dose is missed at school, depending on their place of residence, a young person may receive a free catch-up dose at their school, their local Aboriginal or government health centre, or private general practitioner.

### Immunisation program impacts

In Australia, the prevalence of the HPV types in women aged 18 to 24 years old which were targeted by the vaccine declined significantly from 28.7% in the pre-vaccination era (2005-2007), to 2.3% in vaccinated women after vaccine introduction (2010-2012). ^5^ The rate of high-grade cervical disease has also declined dramatically, by 2014 it dropped to less than half the 2007 rate in women under 20 years of age. ^5^ The introduction of the Gardasil 9 vaccine is expected to prevent up to 90% of cervical cancers. Some research suggests that increased HPV vaccination, together with greater participation in screening, has the potential to eliminate cervical cancer in Australia and elsewhere within a few decades. ^5, 6^

In Australia, young Aboriginal and Torres Strait Islander (hereafter, respectfully, Aboriginal) people do not receive their HPV vaccine dose, resulting in missed opportunities to be protected from HPV-related cancers. ^4^ This is particularly important as rates of cervical cancer in Aboriginal women are twice those of non-Indigenous women, mortality rates more than three times higher, and screening participation rates lower. ^6– 9^ The recent change to one HPV dose will have substantive impact on meeting the recommended dose proposed by WHO. ^4^ In 2022, the Australian national rate of having at least one dose of HPV vaccination for young people turning 15 years old was 83.0% for Aboriginal females and 78.1% for Aboriginal males. ^10^ In Western Australia (WA), the completion rates were 81.4% of Aboriginal females and 78.6% of Aboriginal males. Despite these overall rates being reasonable, there are pockets of concern within Western Australian, including the capital of the State, Perth. In 2022, 51% of Aboriginal young people in Perth received one dose of HPV vaccine during Year 7 and this did not improve in 2023 (unpublished data). ^11^ Substantive resources are needed to improve these rates both in school catch-up programs and within the community. To eliminate cervical cancer, improving HPV vaccine rates amongst young Aboriginal people is necessary.

Despite the decline in HPV, there are a number of reported barriers associated with HPV vaccine uptake including adolescents’ lack of awareness and knowledge, not feeling informed, and parental concerns around sexual activity and vaccine safety. ^12^ Adolescent boys were a ‘neglected’ group in understanding their barriers, with few studies examining the awareness, knowledge and intention of parents to vaccinate their boys. ^12^ COVID-19 has also had an impact on HPV vaccination with a global decline in HPV vaccination rates, particularly for low and middle income countries. ^13^ There is limited information on barriers and enablers regarding uptake of the HPV vaccine amongst global Indigenous communities. ^14, 15^ Acceptance of the vaccine included the provision of health information and school vaccination programs. In contrast, the lack of culturally appropriate information and services was identified as driving hesitancy. The intergenerational disruptions to communities and cultural traditions resulting from colonisation, and distrust of health systems due to histories of disadvantage and exploitation, are described as significant barriers. ^14^ While generalisability is limited, findings from this review echo those from Australian studies. ^6, 16, 17^

### Potential solution

For many young people, ‘[d] igital technology is regarded as an extension of self and social media is a primary mode of communication and social engagement’. ^18^ This extends to an interest in using social media for health-related information. ^18, 19^ A qualitative UK study with 13-18 year-olds found that the relatability and perceived sincerity of YouTube Influencers impacted their acceptance of health messages. Familiarity with the Influencer was also a factor for some younger males. At the same time, other participants were concerned that generalising an Influencer’s personal experiences carried a risk of negative effects. ^20^ Further research has found that tailoring strategies to the target culture, collaborating with community Influencers and ‘trusted messengers’, and leveraging ‘narratives and storytelling’ on various forms of social media to address specific health issues are likely to provide messages with greater reach and persuasive power. ^19, 21^ For young people, there is an increasing body of literature on social media use which cover issues such as substance use, ^22^ flu vaccination, ^23^ and sexual health ^24^ including HPV. Interventions in the general population for HPV vaccination using vehicles such as Facebook, YouTube, Twitter, Instagram ^25– 27^ and TikTok ^28^ have been found useful for enhancing issue awareness and providing information and autonomy in accessing and utilising, and in debunking misinformation.

Aboriginal people are high adopters of using social media platforms. Although data is limited on the use of social media platforms amongst young Aboriginal people, a 2014 survey of 400 Aboriginal participants of all ages found that 60% used Facebook compared to 42% of the Australian population. ^29^ The rise of TikTok has also had a big impact on Aboriginal creators and has fostered a community for young people to talk about their culture. ^30^ There are currently 42.1M posts of short videos related to or delivered by Australian Aboriginal people on TikTok ( https://www.tiktok.com/discover/australia-aboriginals ). Currently, it is the digital place to be for young people, having previously overtaken Snapchat, and is expected to become more popular than X (formally known as Twitter). ^31^ TikTok is the ideal place for a health promotion social media campaign for young Aboriginal people.

Social media Influencer posts are recognised in the literature as a potentially effective way to connect with young people and provide them with evidence-based health-related information. We have been unable to locate a social media campaign led by social media Influencers targeting Aboriginal young people with messaging encouraging them to get their HPV vaccine. Most campaigns that have targeted adolescents are highly scripted, traditionally come from established government or not for profit organisations, do not provide culturally relevant messages and/or are not co-created with young people. They also lack the capacity for young people to engage in the campaign and produce peer-to-peer sharing of information. Campaigns do not appear to consider cultural and other barriers to discussing STIs, sexual health, and the HPV vaccine, which is important when choosing messaging. In addition, utilising the skills of Influencers to engage their followers is likely to improve the acceptance of messages by their target audience.

Therefore, the **overall aim** of this project is to improve the rates of HPV vaccination amongst young Aboriginal people. This will be achieved through the following aims:

*Aim 1:* Co-create health promotion messages with young Aboriginal people and their families.

*Aim 2:* Develop and deliver a social media campaign focused on these messages through micro-Influencers.

*Aim 3:* Evaluate the effectiveness of the campaign through an interrupted time-series design.

We **hypothesise** that the co-creation of health promotion messages with young Aboriginal people and their families and utilising the skills of local micro-Influencers to engage and influence their followers, will improve HPV vaccination rates amongst this population.

## Protocol

### Setting and target population

This public health project will be delivered to male and female young Aboriginal people in the capital city of Western Australia (Australia), Perth. We have concentrated this project in Perth as i) it is not feasible to co-create effective health promotion messages for all regions of Western Australia due to geographic size (2.646 million km ^2^), and ii) given the large population of young Aboriginal people in the metropolitan area, we are able to effectively evaluate the impact of this project. Our primary target population will be young Aboriginal people aged 12-16 years old. The minimum age of involvement in the study is defined by the age that young people receive the vaccine at school and our maximum age is based on one-year post the age that is used for international reporting of HPV vaccination completion (see primary outcome). In 2023, we estimated 4983 young Aboriginal people aged 12-16 years old living in Perth. ^32^

### Collaboration

The project is a collaboration between Edith Cowan University (ECU), and the Aboriginal Health Council of Western Australia (AHCWA). Within AHCWA, the Public Health, Sexual Health and Blood Borne Virus, and Aboriginal Youth Programs will be actively involved in the project. The project will support two Aboriginal young people as ‘health promotion research assistants’ and be supported to formally develop their skills in health promotion.

### Cultural governance group

Given sensitivities surrounding this topic, we will have a Cultural Governance Group that will provide high-level cultural governance. The group will be comprised of the Aboriginal CIs (DM, JJ, KG), the Aboriginal health promotion research assistants, at least one young Aboriginal person aged between 13-15 years old, a family member who has a teenager, an Aboriginal community member and an Elder. The group will provide cultural advice to the social media team on the content and brief for the Influencers, branding and advice and coaching of Influencers.

**Figure 1.**
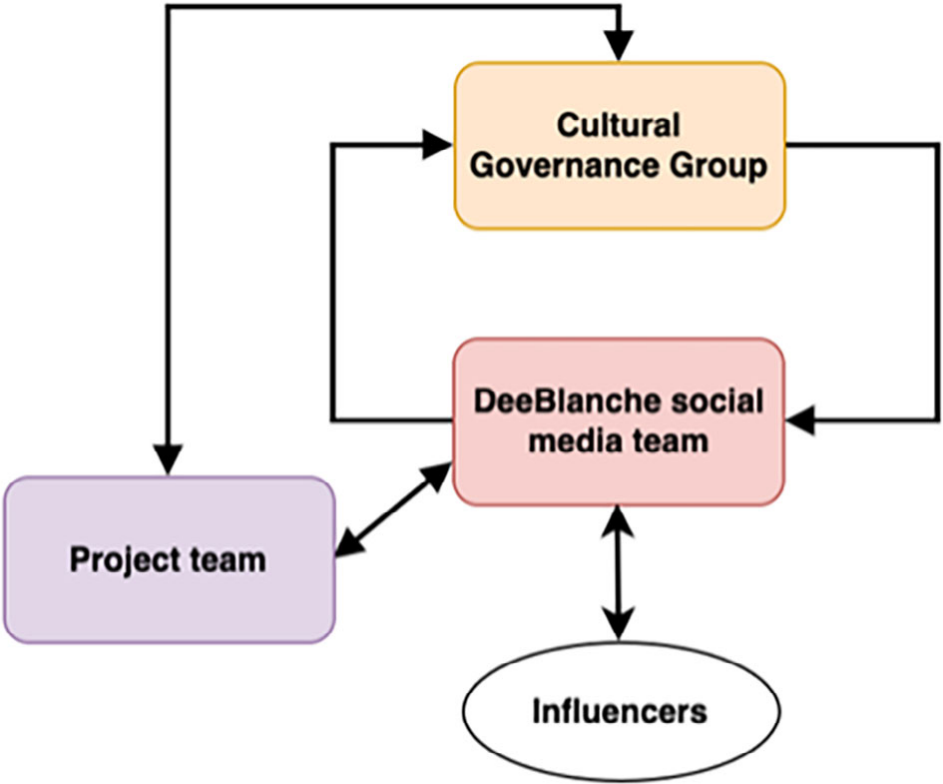
Relationship of cultural governance group to the project and social media team.

They will also provide advice to the project team on question development and engagement of young people and interpretation of results. Figure 1 provides the relationship between the Cultural Governance Group and the project and social media team. The group will meet quarterly in the first year and then biannually in years 2-4.

### Study design

**Figure 2.**
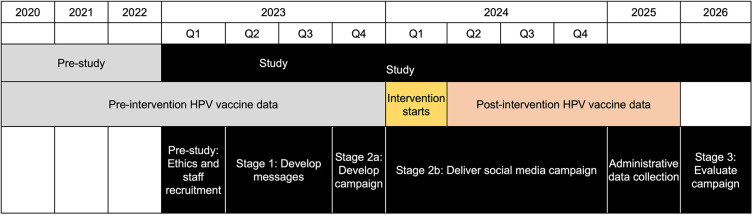
Interrupted time series design for identifying strength-based approaches that are feasible, sustainable and culturally safe for public health initiatives with Aboriginal and Torres Strait Islander peoples.

This project will be delivered in three stages: (i) develop health promotion messages, (ii) develop and deliver a social media campaign, and (iii) evaluate the campaign using an interrupted time-series design. Figure 2 provides an overview of the project including development of the campaign, qualitative and quantitative data collection and evaluation. We will use a controlled interrupted time series (CITS) design to determine the effectiveness of the social media campaign on HPV vaccination rates.

### Stage 1: Develop health promotion messages

*Aim 1: Develop health promotion messages through co-creation with young Aboriginal people and their families*

**Participants and recruitment**

To ensure messages are appropriately targeted to Aboriginal youth, we will invite young people aged 12-16 years old and their families for interviews. For young people, the aim will be to determine their understanding of the HPV vaccine and what messages would influence their uptake of the vaccine. We will also ascertain what Influencers they follow. Families will be encouraged to participate in the interviews as well. Families will be asked about their thoughts and understanding of the HPV vaccine and aspects that have previously (or would in the future) give them the confidence to support their children to receive the vaccine. We will take a multi-pronged approach to engaging young people and their families. We will engage families who have teenagers of this age through social media posts, community centres, and snowballing techniques. Family members and young people will be interviewed separately. Young people aged 12-13 years old will require family consent to participate in the study. Young people aged 14-16 can either consent for themselves or have the parent/guardian consent for them. If they wish to consent for themselves the Gillick Competency Screen will be completed to ensure they meet the criteria for a mature minor. ^33^ The two Aboriginal health promotion research assistants will be instrumental in recruiting young people and their families. We will recruit from 10 high school catchment zones with a high proportion of Aboriginal students attending schools. We will recruit ten young people aged 12-16 years and five families per catchment area to the project, totalling 100 12–16 year-olds and 50 families. Each person interviewed will be provided with a $50 voucher.

**Data collection and analysis**

We will offer individual face-to-face interviews and focus groups. Interviews will be completed by an experienced Aboriginal qualitative researcher who will also take a mentoring role with the health promotion research assistants to undertake interviews. Interviews will be recorded and transcribed. All qualitative information will then be synthesised descriptively, key messages will be identified, with thematic analysis completed using Nvivo software. Messages will also be discussed with AHCWA’s Sexual Health and Blood Borne Virus team and key stakeholders such as members of WA Health to review the messages for accuracy. At the end of this process, a list of facts and messages will have been developed, which will be discussed with the Cultural Governance Group and then utilised in ‘stage 2’ of the project. In addition, we will have developed a list of Influencers to approach.

### Stage 2: Develop and implement a social media campaign

*Aim 2a: Develop social media campaign through micro-Influencers*

We have engaged social media campaign experts from DeeBlanche ( https://www.deeblanche.com/). They are a boutique marketing agency that specialises in developing and rolling out campaign brands for fashion and hospitality, and who have a unique understanding of the social media habits of Generation Z. The team will include experts in strategy development, production, execution, monitoring, reporting and evaluation of the campaign, and will be responsible for:

• **Pre-strategy **: Supporting question development and idea generation of brand.
• **Strategy development:** Understanding information from stage 1, key message development, branding, tone of the campaign, the timing of posts, content and brief for Influencers, and resource management.
• **Strategy implementation:** The roll-out of the campaign, support and recruitment of Influencers, scheduling of Influencer content, account management, reporting of activity/success, campaign reporting, and advice/coaching to Influencers.

Based on the advice from DeeBlanche, we will use TikTok as our platform for young people and Instagram and Facebook for families. For young people, we will engage at least five Aboriginal TikTok ‘micro-Influencers’ who have been identified in stage 1. Micro-Influencers are defined as Influencers who have up to 10,000 followers, have high engagement rates, strong relationships with their followers, and their opinions and perceptions are highly valued amongst their followers. Based on the experience of our social media team, micro-Influencers are the most appropriate type of Influencers to deliver this campaign compared to other types of Influencers (e.g. mega- and macro-Influencers). With the support of our social media team, micro-Influencers will provide content in their style. We will prioritise Aboriginal micro-Influencers, and they will be paid based on their industry rates, and own their own content. They will be required to add in #sponsored or equivalent to their posts. Influencers will not be expected to respond to comments on their posts, however, posts will be monitored by the social media team to provide feedback and mitigate any negative publicity or misinformation. During this stage, we will also develop linktrees that will be used as part of the campaign and will direct young people to relevant health services for catch-up HPV vaccination. For families, we will deliver content targeted to them via Instagram and Facebook as this platform has more engagement with this slightly older demographic. The Instagram and Facebook content will be general advertisement information developed by our social media team based on interviews in stage 1.

*Aim 2b: Implement a social media campaign*

**Figure 3.**
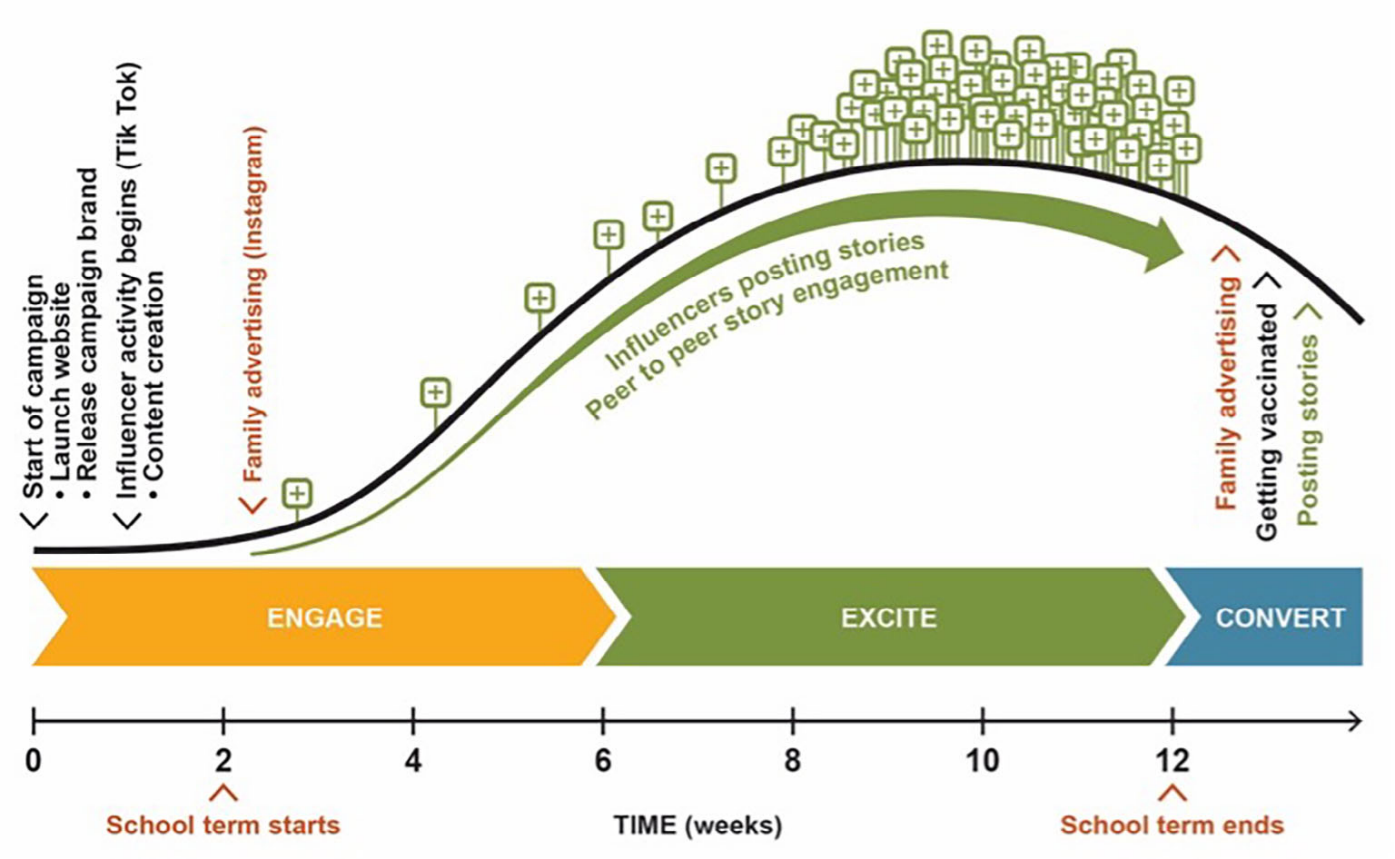
Implementation of campaign during Term 1 and 3.

We will deliver the campaign in Year 2 of the project ( Figure 3). The campaign will start prior to school term 1 and continue on to term 3. The timing of the campaign is to coincide with the school vaccination program, including parent consent and HPV dose 1 and the HPV vaccine catch-up program. Figure 2 provides a social media campaign journey and focuses on the ‘engage ➔ excite ➔ convert’ part of the digital marketing strategy. **‘Engage’** is priming young people about the social campaign for HPV. This will include the release of our campaign brand and launch of the website (weeks 1-6). **‘Excite’** is the main part of delivering the campaign through micro-Influencers. Micro-Influencers will deliver up to 10 posts per week in total on their social media accounts during the build-up period (weeks 6-8), with 20-40 posts per week in total during the peak times (weeks 8-12). We expect this period to include organic content created by young people who are followers of the micro-Influencers and circulated through TikTok. **‘Convert’** will be when young people have access to vaccinations (after week 12). During this period, we expect there will again be peer to peer posts created on TikTok that will keep the campaign’s momentum until the campaign starts again in term 3. Families will be targeted during weeks 2-3 and after week 12 through a content on Instagram.

### Stage 3: Evaluation of the campaign

*Aim 3: Evaluate the effectiveness of the campaign through an interrupted time-series design*

**Controlled interrupted time series (CITS)**

This CITS study will be conducted over 72 months and includes three distinct periods: a 49-month pre-intervention period (2020-2023), a 4-month intervention period and a 19-month post-intervention period (2024-2025). ITS is a quasi-experimental design for assessing public health interventions and is a robust method when other designs such as randomised controlled trials are impractical. ITS study designs allow adjustment of potential biases such as secular trends, seasonal trends, random fluctuations, and autocorrelation compared to other quasi-experimental designs such as controlled before and after studies. ^34^ Inclusion of a control group can strengthen an ITS evaluation by allowing assessment of whether any observed impact of the intervention is likely causal, or could instead be due to co-interventions or other events occurring concurrently with the intervention.

**Intervention and control populations**

The social media campaign will be targeted to Aboriginal young people in the Perth region. The control group will be non-Aboriginal young people in the same location. We have chosen this characteristic-based control group because we expect that any co-interventions or concurrent events would likely affect both groups. ^35^ Therefore, if an impact is observed in both groups, this suggests it may be due to a co-intervention/concurrent event, while if the impact is only observed in the intervention series, this suggests the effect is more likely to be due to the intervention. During the intervention delivery we will collect information on events and interventions that occurred within Perth to improve HPV vaccination rates and who were the target population. We will report these in our final publication.

**Data collection**

Non-identifiable HPV vaccination data from the Australian Immunisation Register (AIR) will be requested from the Australian Institute of Health and Welfare (AIHW)’ National Health Data Hub. ^36^ Data will be requested for Aboriginal and non-Aboriginal people aged 12-25 years during 2020 to 2025 in the Perth region. Data from AIR will also include Aboriginal status, the timing of vaccinations, provider location, date of birth, address postcode and sex.

Analytics and information from Influencers delivering the campaign will also be collected to evaluate the campaign. We will also complete online or telephone interviews with the Influencers and managers of Influencers that have participated in our campaign to discuss the how they created content for the campaign, their ability to influence large audiences, and what worked and what could be improved in the process of working with the campaign. Influencers and managers will be provided with a $50 voucher for their participation in the interview.

**Outcomes**

The primary outcome, as defined by the World Health Organization and for reporting Australian reporting standards as a recipient of the HPV vaccination by the age of 15 years (ie before their 15 ^th^ birthday). ^37^ Secondary outcome will be the number of young people who are the recipient of the HPV vaccination at 25 years old. ^37^

For Influencer analytics we will measure total number views, total likes, comments, and total shares. From our own social media platforms we’ll report on the average time each video was watched, whether a video was watched in full (percentage of viewers who watched the entire video), the source of where people found the videos and where the viewers were located.

**Sample size**

Based on data from the Australian Bureau of Statistics, we anticipate approximately 89 Aboriginal youth turning 15 years old each month in Perth over the study period. ^32^ Using the primary outcome definition, monthly numbers of young people before their 15 ^th^ birthday fully vaccinated against HPV, will be the unit of aggregation in the regression models. A total of 72 monthly data points (2020-2025) will provide 85% power to detect a one-standard-deviation shift in the average proportions post-intervention, at an α = 0.05 and a first-order autoregressive model with autocorrelation of 0.3. ^38^ Empirical research has shown autocorrelations of the order of 0.3 (median 0.33; IQR 0.03, 0.58; 36 ITS studies in public health). ^39^

**Data analysis**

*Interrupted time series*

A linear, segmented interrupted time series analysis will be used to evaluate the impact of the social media programme on HPV vaccination.

For the primary outcome, we will count the number of youths aged between 12 and before their 15 ^th^ birthday who have received the HPV vaccination per month. Similarly, for the secondary outcome, we will count the number of young people aged up to 25 (inclusive) who have received the HVP vaccination per month. Our analysis models will include two segments: a pre-interruption period of 49 months, beginning on Jan 2020-Dec 2023, and a post-interruption period of 24 months, beginning on Jan 2024-Dec 2025, which encompasses both the intervention and post-intervention periods.

For each outcome, we will fit the following statistical model (Model 1), separately for the intervention and control series:

$$\text{Model}\;1:\log Y_{t}=\beta_{0}+\beta_{1}t+\beta_{2}I+\beta_{3}\left(t-T_{I}\right)I+\text{seasonal}+\text{offset}+\epsilon_{t}$$

where $Y_{t}$ is the outcome value at time t, and $T_{I}$ is the time of the interruption. The variable I indicates the post-interruption segment, which is equal to 1 post the interruption and 0 before. Parameter $\beta_{0}$ is the intercept at the beginning of the series, $\beta_{1}$ is the slope in the first segment, $\beta_{2}$ is the level change between the expected value (based on the pre-interruption data) and the observed data, and $\beta_{3}$ is the difference in slope compared to $\beta_{1}$ . The error term, $\varepsilon_{t}$ represents deviations from the fitted model, and we will assume these to have lag-1 autocorrelation. Seasonality will be accounted for by including two pairs of sine and cosine harmonics with periods of 12 and 6 months. ^40, 41^ Due to the changing of the vaccine schedule in the year following the interruption, the seasonal components will be phase shifted by six months for that year. The log of the relevant population (i.e. all Aboriginal youth aged between 12 and before their 15 ^th^ birthday per month) will be included as an exposure offset to adjust for any changes to the population over time. We will assume a negative binomial distribution with a log link function. We will fit these models using the glarma package in R. ^42^

The effect measures of interest will be:

• Level change at the end of the social media campaign (i.e. three months post the beginning of the social media campaign).
• Change in slope.

In addition to the above model, for both outcomes, we will also fit the following single model (Model 2) that incorporates both the intervention and control series:

$$\text{Model}\;2:\log Y_{t}=\beta_{0}+\beta_{1}t+\beta_{2}I+\beta_{3}\left(t-T_{I}\right)I+\beta_{4}G+\beta_{5}\mathrm{tG}+\beta_{6}\mathrm{IG}+\beta_{7}\left(t-T_{I}\right)\mathrm{IG}+\text{seasonal}+\text{offset}+\epsilon_{t}$$

where $Y_{t},T_{I},I,t,\text{seasonal},\text{offset and}\;\epsilon_{t}\;$ are as described above. G represents the series with a value of 1 representing the intervention series, and a value of 0 representing the control series. $\beta_{0}$ , $\beta_{1}$ , $\beta_{2}$ and $\beta_{3}$ are as described above for the control series. $\beta_{4},\beta_{5},\beta_{6}\;\text{and}\;\beta_{7}$ represent the interactions between time and the intervention series. Specifically, $\beta_{4}$ represents the difference in the intercept at the beginning of the series between the intervention and control series, $\beta_{5}$ represents the difference in slope between the intervention and control series in the first segment, $\beta_{6}$ represents the difference in the level change between the intervention and control series, and $\beta_{7}$ represents the difference in the change in slope between the intervention and the control series.

We will compare the estimates of level change at the end of the social media campaign and change in slope between Model 1 and Model 2. This comparison will allow us to assess the possibility of whether a co-intervention/concurrent event may explain our findings. ^35^

We will plot interrupted time series graphs for all outcomes and series using the recommendations of Turner et al. ^43^ These plots will include: data points, trend lines, seasonality estimation and confidence intervals surrounding the trend line and counterfactual estimates. Segments will clearly delineated by breaks in the trend lines.

Subgroup analysis

We will fit Model 1 for the primary and secondary outcomes for the intervention series separately by:

• Sex (Females, Males)
• Location in Perth (North West, North East, Central, South West and South East)

Influencer data

All qualitative information will then be synthesised descriptively, key messages will be identified, with thematic analysis completed using Nvivo software.

### Dissemination

Part of the outcomes of this project will be a brand that will be associated with the HPV vaccine. It is expected this brand will organically be distributed amongst Influencers and their followers. We will also circulate the brand to health services and other organisations through collaborations with Aboriginal organisations. If this model is successful in improving HPV vaccination rates, the process of creating these improvements, the development of a public health model that engages young Aboriginal people to improve their health outcomes, will be easily translatable to other Aboriginal communities, health outcomes and populations. We will have developed a step-by-step guide on how to complete this, including branding that can be used by any young person or Influencer.

### Study status

Intervention started Feb 2024.

## Discussion

This project aims to improve HPV vaccination rates amongst young Aboriginal people. Nationally, 75% of Aboriginal females and 68% of Aboriginal males receive their full course of the HPV vaccine at 15 years of age, however, there is substantial variation between states and territories. Given that the vaccine reduces the risk of cervical cancer, which has a high mortality rate in Aboriginal women, improving HPV vaccine rates is essential. Our project will combine health messages co-created by young people and implemented by micro-Influencers to improve HPV vaccination. Our vision is to develop campaign that can be implemented by other Aboriginal communities, settings and populations across Australia to improve HPV vaccination.

## Ethical considerations

Approvals were obtained from the Western Australian Aboriginal Health Ethics Committee (WAAHEC Ref No: 1227) and Edith Cowan University (2022-03993 STROBEL). This study adheres to the Declaration of Helsinki.

## Consent to participate

Consent was received for all interviews conducted all participants. Interviews with young people and families required written consent. Interviews with influencers required verbal. Verbal consent was approved as Influencers are located around Australia and we were unable to complete face to face interviews, therefore online interviews were completed. Verbal consent for these was ethically approved. Ethics has been approved for all consent processes described. For HPV vaccine data a waiver of consent was approved to receive these data.

## Data Availability

No data is associated with this article. We will provide full PICFs and interview guides on request and if approved by our governance group. We have provided the SPIRIT guidelines in Figshare ( https://doi.org/10.6084/m9.figshare.29833178.v5) ^45^ and amended as needed for this study design. ^44^ We have also provided an example PICF and interview guide. We will provide full PICFs and interview guides on request and if approved by our governance group.
