# Supplementary material for: IMPROVING HPV VACCINE RATES FOR ABORIGINAL YOUNG PEOPLE THROUGH A SOCIAL MEDIA CAMPAIGN: AN INTERRUPTED TIME SERIES
Source: F1000Res. 2025 Oct 24;14:1162. [Version 1] doi: 10.12688/f1000research.168930.1 (PMC12789854; doi:10.12688/f1000research.168930.1)
Supplement: Peer review history

# Reviewer report: Reviewer response for version 1

**DOI:** 10.5256/f1000research.186158.r448485

**Published:** 2026-01-10

**Authors:** Namoonga M Mantina

**Affiliations:** 1 University of Arizona, Arizona, Tucson, USA

I advise the authors indicate in the title that this is a protocol, just incase it is not immediately evident from the  journal's typeface (It wasn't immediately evident to me as a reviewer that this was a protocol paper)

 How many micro-influencers to do you anticipate engaging?

 It is unclear to me why the secondary outcome pertains to recipients of the vaccine at age 25, when the bulk of the manuscript speaks to adolescents age 12-16years and these are stated to be the target population. What is the significance of this in relation to the study? Some context to this would be helpful

Is the study design appropriate for the research question?

Yes

Is the rationale for, and objectives of, the study clearly described?

Yes

Are sufficient details of the methods provided to allow replication by others?

Yes

Are the datasets clearly presented in a useable and accessible format?

Not applicable

Reviewer Expertise:

HPV vaccination, community engagement

I confirm that I have read this submission and believe that I have an appropriate level of expertise to confirm that it is of an acceptable scientific standard.

# Reviewer report: Reviewer response for version 1

**DOI:** 10.5256/f1000research.186158.r429973

**Published:** 2025-11-25

**Authors:** Oluwakemi Alonge

**Affiliations:** 1 Marshfield Clinic Research Institute, Marshfield, Wisconsin, USA

This study aims to evaluate HPV vaccination uptake among aboriginal adolescents through a social media HPV campaign. The effectiveness of the campaign will then be evaluated using a time-series analysis. The study design described is suitable for the research question and is highly effective for meeting the study objectives.  The study outcomes are described in detail. The sample size is sufficient to detect a true effect and allows for reliable statistical analysis. I look forward to reading the results from this study.

Is the study design appropriate for the research question?

Yes

Is the rationale for, and objectives of, the study clearly described?

Yes

Are sufficient details of the methods provided to allow replication by others?

Yes

Are the datasets clearly presented in a useable and accessible format?

Yes

Reviewer Expertise:

Infectious diseases, vaccine effectiveness, vaccine promotion

I confirm that I have read this submission and believe that I have an appropriate level of expertise to confirm that it is of an acceptable scientific standard.
